# Stable Gender Gap and Similar Gender Trend in Chronic Morbidities between 1997–2015 in Adult Canary Population

**DOI:** 10.3390/ijerph19159404

**Published:** 2022-07-31

**Authors:** Luis Miguel Bello-Lujan, Jose Antonio Serrano-Sanchez, Juan Jose Gonzalez-Henriquez

**Affiliations:** 1 Research Group of Human Performance, Physical Activity and Health, University of Las Palmas, Las Palmas de Gran Canaria, 35017 Canarias, Spain; lbelluj@telefonica.net; 2 Research Institute of Biomedical and Health Sciences (IUIBS), Las Palmas de Gran Canaria, 35005 Canarias, Spain; 3 Department of Mathematics, University of Las Palmas de Gran Canaria, Las Palmas de Gran Canaria, 35017 Canarias, Spain; juanjose.gonzalez@ulpgc.es

**Keywords:** expansion, chronic morbidity, gender, disparities, aging

## Abstract

There is little information about the trend of the gender gap in chronic morbidities and whether the trend of expansion occurs equally in the age and gender groups. The objectives were to examine the consistency and stability of the gender gap in the main self-reported chronic morbidities in the general population, and, likewise, to analyze the trend of major chronic morbidities between 1997 and 2015 in men and women across age groups. The data were extracted from the Canary Health Survey, which uses a probabilistic sampling in the population >16 years of age, for the years 1997 (*n* = 2167), 2004 (*n* = 4304), 2009 (*n* = 4542), and 2015 (*n* = 4560). The data for the twelve most frequent chronic morbidities were analyzed using logistic regression, estimating the annual change ratio between 1997 and 2015, adjusting for age and educational level. The interaction of age with the period (1997–2015) was examined to analyze the rate of change for each morbidity in the age groups. Musculoskeletal diseases, headaches, anxiety and depression, and peripheral vascular diseases showed a stable gender gap across observed years. High cholesterol and high blood pressure tended to a gap reduction, while heart disease, diabetes, and respiratory disease did not show a significant gender gap along the period. The trend of the main chronic morbidities increased similarly in men and women in all age groups, but significantly in women older than 60 years and in men older than 45 years. Aging explained a substantial part of the trend of increasing prevalence of the main chronic morbidities, but not totally. Factors other than age and education are driving the increase in chronic morbidity in older age groups.

## 1. Introduction

Chronic morbidity is one of the most serious public health problems due to its high prevalence and cost to health systems [1,2,3]. Approximately 50% of the healthy life years lost in the world population are attributed to chronic diseases [4]. In combination with COVID-19, some chronic diseases, such as diabetes and cardiovascular diseases, produce a substantial deterioration in health, increasing the risk of death and critical care [5,6].

Chronic morbidity is understood as a diagnosed disease or medical condition which either requires continuous, long-term medical attention or limits daily life activities, or both [7]. Studies on gender differences in chronic morbidity reflect a differential pattern between men and women [8,9,10]. An almost 5-year gap of life with disability has been reported in women, with the main contributors being a lower mortality and a higher prevalence of functional limitations compared to men [9]. Women tend to report worse health in a variety of chronic conditions, particularly musculoskeletal diseases such as arthritis and osteoporosis [11,12], respiratory diseases such as asthma, bronchitis, and severe degrees of chronic obstructive pulmonary disease [13,14,15], migraines and headaches [13], depressive disorders and anxiety [9,13], and peripheral circulatory problems such as varicose veins [10]. Men tend to suffer from fewer chronic morbidities, but more fatalities from, for example, heart disease, arteriosclerosis, and emphysema [10].

The studies of gender inequalities in health have shown different results depending on the health measure used [16,17,18]. Relatively stable results in the unfavorable gender gap for women have been reported in the area of chronic morbidity with functional limitations, with a tendency to increase the gender gap with age [8]. Likewise, in the management of some diseases, such as acute stroke, it was observed that women presented greater disability and poorer results than men after 3 months of follow-up [19,20]. Few studies have analyzed whether the gender gap is constant over time for the most prevalent chronic morbidities. It is argued that gender inequalities in health are a consequence of an underlying social stratification that mediates exposure to harmful health risks and access to goods that promote well-being [21,22,23]. The gender gap in chronic morbidity might not be constant over time and this would affect the consistency of gender differences. In this study, we examine whether the differences between men and women in the prevalence of the most common chronic morbidities remained constant between 1997, 2004, 2009, and 2015.

The advance of chronic morbidity in developed societies is a process that has been observed since the middle of the last century and has been attributed mainly to an increase in life expectancy [24,25,26,27] and a shift in lifestyle toward greater sedentary behavior, poor diet, and other unhealthy behaviors [28,29,30,31]. Although there is no scientific consensus on the development model of chronic morbidity, the expansion theory gathers a greater amount of evidence for the increase of unhealthy life years [32,33,34,35], also in Spain [36,37]. Some differences between men and women in the morbidity trend have been reported in Spanish adults over 65 years of age, with men showing an expansion of morbidity, defined as a decrease in disease-free life years, while women experienced an increase in disease-free life years in about half of the different Spanish regions [38]. There is little information about whether the trend of specific chronic morbidities may differ by age. Hence, we analyze the interaction of age with the period of time analyzed (1997–2015) to examine whether some age groups are expanding at a different pace.

Monitoring the trend in the prevalence of chronic morbidities in the general population is important to detect changes that allow for the implementation of appropriate care policies and to reduce health inequalities. Chronic morbidities are the main contributors to disability in men and women [39], which directly affect the individual quality of life and requirements of medical care and medication, driving the level of use of health resources.

Thus, the present work has two main objectives. The first is to analyze the presence of a possible gender gap in the most prevalent chronic morbidities and its stability across time (1997, 2004, 2009, and 2015) in the adult general population. We hypothesize that we will find a stable female gap over time for some chronic morbidities, particularly musculoskeletal, mental, respiratory diseases, and headaches. The second objective is to examine the trend in the prevalence of the main chronic diseases between 1997 and 2015 at the population level and according to age and gender groups. In a context of expanding morbidity, the hypothesis is that all chronic morbidities observed are increasing in their prevalence in the general population, with the trend being faster in older age groups, which is consistent with the expansion theory of morbidity [40,41].

## 2. Materials and Methods

We used a repeated cross-sectional survey with four independent samples from the Canary Health Survey (CHS), a periodic probabilistic survey (5–7 years) in the census population ≥16 years old [42,43]. In a repeated cross-sectional design, there may be zero overlap in the samples between periods, and yet valid inferences of change in population values can be made on the basis of repeated cross-sections [44,45]. This study uses the surveys conducted in 1997 (*n* = 2176), 2004 (*n* = 4320), 2009 (*n* = 4560), and 2015 (*n* = 4578).

### 2.1. Study Design and Participants

The participants were selected trough a multistage sampling, stratified by island, geographic region, municipal size, and socioeconomic level of the census sections. Within each census section, the households and participants were randomly selected, maintaining a proportional distribution by age group and gender. Since 2004, nonproportional allocations have been made in the group of women over 60 years of age, assigning between 400–500 additional surveys to obtain more precise estimates of this group.

The participants were notified, via letter of the selection, the date of the visit and the legal provisions that regulate participation in the CHS. All participants were interviewed at their place of residence. In 2004, the CAPI (computer-assisted personal interview) system was incorporated, and the interviewers were equipped with a tablet computer that contained the corresponding questionnaire and the digital cartography that included the randomly selected households. The participants were informed of the objectives and their verbal consent was requested. The response rate was 91–95% of the selected dwellings. Between 5–9% of the interviews conducted by the CHS were reassigned to the household adjacent to the original upon three failed contact attempts or upon the original participant’s refusal to be interviewed. Additional and detailed methodological information can be found in the report published by the ISTAC [40].

### 2.2. Chronic Morbidity

Data on the positive cases of chronic morbidities were obtained using the CHS adult questionnaire in personal interviews [46]. The questionnaire collects information about 28 level-3 chronic morbidities in the Global Burden Diseases (GBD) protocol, defined as those greater than 6 months in duration, and diagnosed by a doctor within the previous 12 months. The participants were prompted as follows: “I am now going to list a series of chronic or long-standing diseases so that you can tell us whether you currently suffer or have ever suffered from any of them. By long-standing we mean that the health issue has lasted or is expected to last six months or longer”. For each and every morbidity that received an affirmative response, two follow-up questions were subsequently asked to the participants: whether they had suffered it in the previous 12 months, and whether it had been formally diagnosed by a physician. In this study, those who answered affirmatively to the three questions were recorded as positive cases of chronic morbidity.

We selected the twelve most frequent chronic morbidities: high blood pressure, high cholesterol, chronic neck and back pain, anxiety and depression, joint–rheumatic pain, chronic digestive diseases, heart diseases, peripheral vascular diseases, diabetes, respiratory diseases, chronic headaches, and osteoporosis. Respiratory diseases include chronic bronchitis, asthma, emphysema, and chronic obstructive pulmonary disease. Heart diseases include angina pectoris, heart attack, and heart failure. Digestive diseases include heartburn, stomach pain from gastritis, and stomach or duodenal ulcers. Peripheral vascular diseases include varicose veins and poor circulation. The two questions about back pain due to lumbago, sciatica, and herniated disc and chronic cervical back pain were combined into a single category, named neck and back pain. It has been shown that the trend of specific chronic morbidities may differ according to the level of aggregation used [22]. Given the relationship between various chronic morbidities, in this study we used two levels to assess cardiovascular and musculoskeletal diseases as separate groups of morbidities. Cardiovascular morbidities included heart diseases, high blood pressure, peripheral vascular diseases, high cholesterol, and stroke. Musculoskeletal morbidities included neck and back pain, joint–rheumatic pain, and osteoporosis.

Several chronic morbidities were excluded from the analysis due to their low prevalence in the general population and the fact they present wide margins of error, below zero, which affects both the standardization in various age or gender groups and the analysis of the trend, with inconsistent results. The morbidities excluded were dementia, malignant tumors, stroke (included in a second level of cardiovascular morbidities), chronic skin problems, urinary incontinence, chronic constipation, hemorrhoids, anemia, thyroid problems, mental retardation, cataracts, allergies, and chronic insomnia. The twelve morbidities selected represented 91.9% (1997), 97.5% (2004), 95.9% (2009), and 96.3% (2015) of those participants who reported at least one chronic morbidity of any kind.

### 2.3. Data Analysis

Participants with missing data in the variables analyzed (between 0.2–0.5%) were excluded after testing for possible differences between men and women (*p* > 0.05) and age groups (*p* > 0.05). The final samples of participants were *n* = 2167 (1997), *n* = 4304 (2004), *n* = 4542 (2009), and *n* = 4560 (2015). For the standardization of the prevalence and its confidence interval (95%), the direct method was followed as previously described [47], taking as the standard the age and sex of the population ≥16 years of age in 2015, as follows: 16–30 years old = 181,477 women and 181,525 men; 31–45 years old = 273,161 women and 280,808 men; 46–60 years old = 236,477 women and 241,203 men; and >60 years old = 221,982 women and 189,025 men. We use 2015 as reference population because it is the most current and there are no large differences between the age distributions in the years analyzed ([48], p. 79).

The prevalence of each morbidity standardized by age was estimated in men and women separately in the four periods analyzed, 1997, 2004, 2009, and 2015. The χ^2^ test was used to calculate *p*-values for gender differences in the prevalence. Differences between men and women in the age-standardized prevalence were expressed as the prevalence ratio (age-standardized prevalence of women/age-standardized prevalence of men) with their respective confidence intervals [49]. Differences across the years were pairwise tested with a two-sample differences z-test of log of prevalence ratios (with Holm–Bonferroni correction) to examine whether potential differences in the prevalence ratio are significantly increasing or decreasing. The results are expressed with superscript letters, assigning the same letter to age groups that did not express significant differences (*p* > 0.05) and different letters to the significantly different groups (*p* < 0.05). The stability of the gender differences across time was estimated with a 5-point scale (0–4), the value 0 representing the absence of significant differences between men and women in the four years analyzed, and the value 4 being the maximum stability of the gender differences in the prevalence of a specific morbidity, with significant differences in the four years observed.

To examine the trend in the prevalence of chronic morbidities over time, we followed the analysis strategy previously suggested [50,51]. We used multivariate logistic regression to estimate the rate of change (odds ratio per year) of morbidities, taking as the dependent variable a chronic morbidity (chronic morbidity = 1, nonchronic morbidity = 0), introducing as covariables the year as a continuous variable, defined as time, the age group (1 = 16–30, 2 = 31–45, 3 = 46–60, and 4 = > 60), and the educational level (1 = primary or lower, 2 = secondary, and 3 = university). We verified the hypothesis of linearity of the variable time in the logit scale with the Box–Tidwell test (adding the interaction term time * ln [time]) [52]. In all cases, the interaction terms were not significant, and therefore it makes sense to treat it as a continuous variable in logistic regression and to analyze the trend by year in the period 1997–2015. To examine whether the trend of the chronic morbidities analyzed differed between the age groups, we performed a second analysis by adding the interaction term age group * time to the previous analysis. In accordance with Jaccard [53], the regression coefficients were calculated by adding the coefficient of the global model for the entire population that corresponded to the age group * time interaction term. The resulting odds ratio expresses the annual change from 1987 to 2015 for each age group ([53], pp. 30–34).

The differences between the age groups in the rate of change of a given morbidity were adjusted with the Holm–Bonferroni multiple comparison test. If some chronic morbidities had advanced further in some age groups, the analysis with the interaction term should reflect a significantly different rate of change between those groups. The data were analyzed with the program R [54,55].

## 3. Results

### 3.1. Descriptive Analysis of Participants

Table 1 shows the changes in the structure of age, gender, education, and occupational activity of the participants in the CHS between 1997 and 2015. A progressive decrease in the relative weight of the 16–30 age group was observed along the years in both men and women. The 31–45 age group remained stable across time and gender, and the oldest age groups (46–60 and >60 years) increased between 8–11% in men and women. The mean age of the participants increased through the periods (from 43.1 to 51.9 years of age in women and from 41.8 to 50.2 in men). Likewise, changes were observed in the educational levels of the participants, with a significant reduction, up to almost half, of those with a primary education or lower, and an increase of double or more in participants with university studies, similar in men and women.

To examine whether such temporal changes could have a sample bias, we compared them against the structure of age, gender, and education using census data (Figure S1). We found great similarities between the CHS and the census data in the structure of age and education, with the exception of gender, motivated by the over-assignment of surveys in the group of older women (>60 years). The segregation of the analyses of men and women and the adjustment for age and education make it possible to standardize the comparisons between the four periods analyzed.

### 3.2. Gender Differences in the Prevalence of Chronic Morbidities

**Figure 1:**
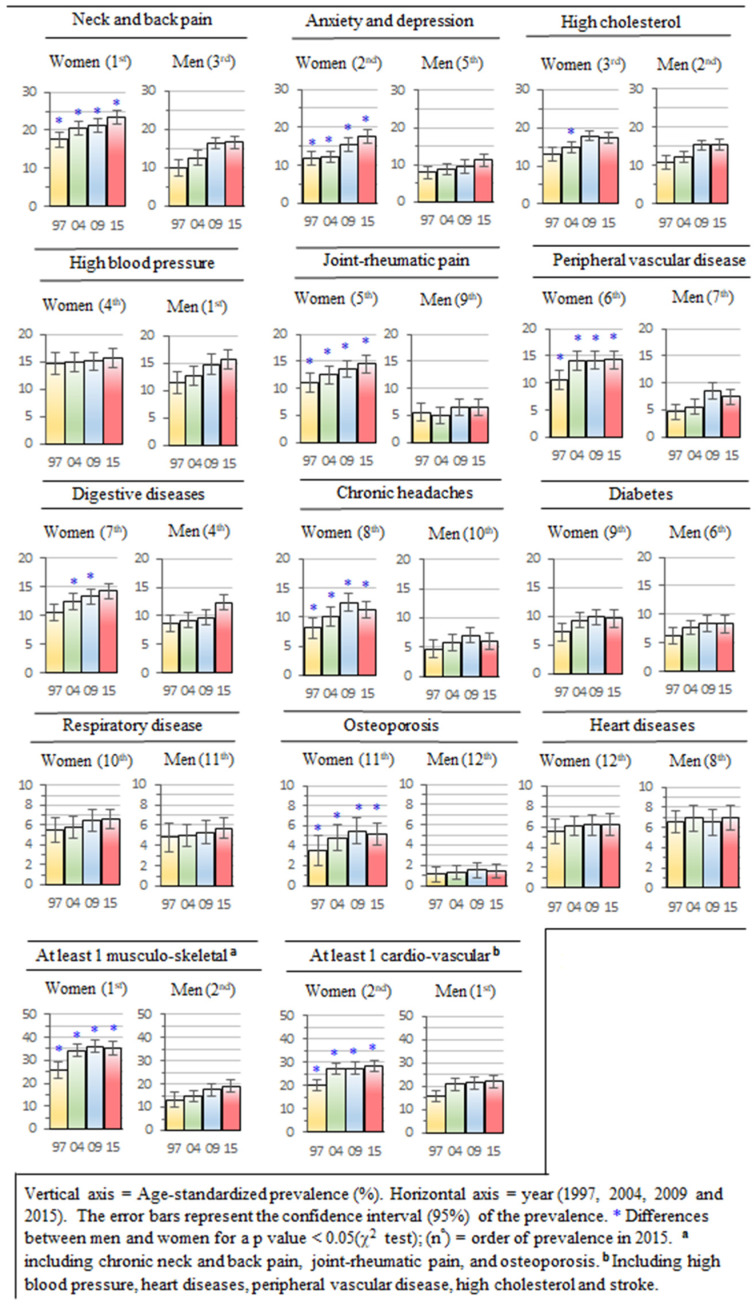
Prevalence of selected chronic morbidity in the Canary Health Survey (1997, 2004, 2009 and 2015).

Figure 1 shows the age-standardized prevalence of the chronic morbidities analyzed. The results are shown in Z order following the prevalence of women in 2015. In general, women had a higher prevalence than men in all the chronic morbidities analyzed, except heart disease. The consistency of the differences between men and women was variable depending on the morbidity analyzed. Table 2 shows the magnitude of differences in relative terms (prevalence ratio) to control for wide differences in the prevalence of morbidities, which led to the use of four different scales in Figure 1 to graphically represent the age-standardized prevalence.

A group of six chronic morbidities showed a significant (*p* < 0.05, χ^2^ test) and stable (in the four years) gender gap, with relative differences between 1.40 and 3.69 of a greater prevalence ratio for women: osteoporosis, joint–rheumatic pain, peripheral vascular disease, chronic headaches, anxiety and depression, and neck and back pain. Chronic digestive diseases and high cholesterol showed moderate or low stability (one or two years). In high blood pressure, diabetes, and respiratory diseases, no significant gender differences were observed in any of the four periods analyzed. The only morbidity where men showed an unfavorable gap was heart diseases, but not significantly (*p* > 0.05).

Considering musculoskeletal and cardiovascular morbidities as aggregated groups (second level), a gender gap was noted between men and women, which was consistent for musculoskeletal morbidities in stability (all years) and magnitude (almost double or more of women with at least one musculoskeletal morbidity). The cardiovascular morbidities showed smaller gender differences, with a prevalence ratio greater in women of between 1.27–1.31 (*p* < 0.05).

The multiple comparison analysis between the years did not generally show consistent variations in the gap for most of the morbidities analyzed, except for peripheral vascular disease and aggregated musculoskeletal morbidities, both with a gap reduction in 2009 and 2015 vs. 2004. The rest of the morbidities did not show significant gender differences between years, although a nonsignificant trend toward a decrease or dilution of the gap could be observed in high cholesterol and high blood pressure.

### 3.3. Trend in the Prevalence of Chronic Morbidities for the Overall Sample

The first two columns in Table 3 show the rate of change of the trend in self-reported chronic morbidities for the overall sample, 1997–2015. Results are shown crude and adjusted by age group and educational level. After adjusting, the rate of change for all morbidities decreased markedly, and for several morbidities it was diluted. Three morbidities in women (respiratory diseases, high blood pressure, and heart diseases) and five morbidities in men (joint–rheumatic pain, respiratory diseases, heart diseases, headaches, and osteoporosis) lost the significance of their ratio of change after adjusting for age and education. The rest of the morbidities decreased in their *p*-value after adjusting for age and education. Age was the main contributor to this decrease, explaining more than 90% of the coefficient of determination R2. Nevertheless, 11 morbidities in women and 9 in men remained with a significant change ratio, showing an increase in the annual odds of these morbidities between 1.5% (joint–rheumatic pain) and 2.8% (osteoporosis) in women and 1.7% (neck–back pain) and 2.8% (high blood pressure) in men after adjusting for age and education.

The two aggregated morbidities analyzed, musculoskeletal and cardiovascular, maintained their consistency after adjusting for age and education, but reduced their annual rate of change by approximately half, up to 2.0% (musculoskeletal) and 2.6% (cardiovascular) of odds annual increase in women and 2.2% (cardiovascular) and 2.5% (musculoskeletal) in men.

### 3.4. Trend of Prevalence in Chronic Morbidities for Age Groups

The third to sixth columns in Table 3 show the annual OR of change in self-reported morbidities for each age group after adding the interaction term “age group by time” in the previous analysis. The results showed age groups–time interactions for some chronic morbidities in both men and women. In women, most of the morbidities showed a significant rate of change for the oldest group (>60 years). In the rest of the female morbidities (headaches, respiratory diseases, and heart diseases) and age groups, no significant differences were observed in the rate of change, except for anxiety and depression in the 46–60 age group.

In men, the trend of seven morbidities (high blood pressure, high cholesterol, neck–back pain, digestive diseases, anxiety and depression, diabetes, and peripheral vascular diseases) expressed an interaction with age, showing a significantly greater rate of change in one or both of the two oldest age groups (from 3.4% to 5.1% of annual increase in their odds ratio). In the rest of the morbidities, no significant changes were observed, despite a concentration trend in the higher rates of change in the older age groups, except for chronic headaches.

The trend of aggregated cardiovascular morbidities showed an interaction with age in the period analyzed, both in men and women. The oldest age groups increased their rate of change significantly in cardiovascular morbidities in women (OR = 1.045, *p* < 0.05) and men (OR = 1.034 for 46–60 years and OR = 1.037 for >60 years, both *p* < 0.05). The aggregated musculoskeletal morbidities followed a similar pattern to the previous one, increasing significantly in the group of women >60 years of age, whilst in men, middle-aged (46–60) and older adults (>60 years) also showed significant rates of change.

## 4. Discussion

The main findings in relation to the gender gap were that men and women presented wide and stable differences in the self-reported prevalence of some chronic morbidities. Regarding the trend, the study found a faster expansion of various chronic morbidities in older age groups, earlier in men than in women.

### 4.1. Gender Gap in Chronic Morbidities

Men and women did not differ much in the five most prevalent chronic morbidities, which in women were neck–back pain, anxiety and depression, high cholesterol, high blood pressure, and joint–rheumatic pain. Men coincided in four of the top-five groups with women except joint–rheumatic pain, which was replaced with digestive diseases in men. Gender differences in the type of morbidity have been analyzed in several studies, which showed women having an excess of morbidities related to functional limitations, disability, joint and muscle pain, and, in general, musculoskeletal diseases that affect mobility, as well as symptoms of depression. However, men have presented an excess of more fatal diseases such as heart and cardiovascular problems [9,11,56], although this excess in fatal morbidity in men has been discussed [8,18].

In our study, the differences between men and women were more ones of degree and temporal stability. The widest and most stable differences resided in musculoskeletal morbidities such as joint–rheumatic pain, osteoporosis, and neck and back pain, as well as in peripheral vascular diseases, chronic headaches, and anxiety and depression, with significant relative differences between genders, specifically a higher prevalence ratio of between 1.40 and 3.69 (*p* < 0.05) in women with respect to men. Digestive diseases presented a smaller and less stable gender difference, between 1.35 (*p* < 0.05) and 1.37 (*p* < 0.05) times higher in women in two of the four years analyzed. High cholesterol showed a weak gender gap of about 1.23 (*p* < 0.05) greater in women in only one of the four years analyzed (2004). No conclusive results were obtained for high blood pressure, diabetes, and respiratory diseases, although women tend to report them more frequently than men. The only morbidity where men presented an unfavorable gap was heart diseases, but it was not significant. The results obtained for respiratory diseases, diabetes, and high blood pressure are contrary to expectations, though they are consistent with the study of European adults >50 years, which found no gender differences in respiratory diseases and diabetes [56].

Although the differences between men and women were consistent, our study failed to capture significant differences over the years that would suggest any alteration in the gap, except for peripheral vascular diseases and aggregated musculoskeletal morbidities, which presented a significant, slight gap reduction in 2004. High cholesterol, high blood pressure, and digestive diseases showed a dilution trend in the gender gap. Given that the comparisons between years are sensitive to the margins of error in the estimates, it is possible that larger samples could offer more precise comparisons that would capture smaller differences in a multiple comparisons test.

Our study provides support to confirm a gender gap with some variability, albeit stable over time, centered on musculoskeletal morbidity (osteoporosis, neck and back pain, joint–rheumatic pain), depression and anxiety, chronic headaches, and peripheral vascular diseases. In the musculoskeletal diseases group, the relative gap in women was more than double that of men, and the main contributors were osteoporosis and joint–rheumatic pain. In the cardiovascular diseases group, the unfavorable gap for women was due to the excess in peripheral vascular diseases and, to a lesser extent, due to high cholesterol and high blood pressure. Other studies reflect a similar differential pattern in chronic morbidity in women. In American women between 45–84 years (1986–2001), a significant excess of headaches, arthritis, depression, hypertension, other pain, and respiratory diseases was found, with values of between 0.5 and 13.2 percentage points [13]. The differences with respect to our study in respiratory diseases and hypertension could be due to the different cut-off point in age. Additionally, Gorman et al. [8] found an excess of functional limitations in women (18–85 years), with the gap increasing with age after adjusting for ethnic, socioeconomic, and health behaviors. However, the prevalence of life-threatening medical conditions did not express clinically relevant gender differences between men and women, except for the faster increase in the gap with age in men [8], showing a related gender gap variability with age.

One of the main causes that can explain the excess of chronic morbidity in women is a higher life expectancy than men and a lower mortality ratio [9,57]. Women are more exposed to chronic morbidity in the additional years of life compared to men. In the Canary population, the life expectancy at birth of women was 7 years higher than men (1997), with a tendency to decrease (5 years in 2015) [58]. The mean age of death increased by 5.3 years in men (from 67.7 to 73.0) and by 3.1 years in women (from 75.8 to 78.9). It would be expected that as the gender gap in mortality narrows, the gender gap in some morbidities will also tend to equalize, although there is no evidence for this hypothesis. A longitudinal study (1985–2004) on gender differences in adults (35–55 years), using various health measures, including various chronic morbidities, showed that women had an excess in some health measures and men in others, but that the association between morbidity and subsequent mortality was similar in both, placing the male–female health–survival paradox into question [18].

An earlier age of onset of chronic morbidity in women could be a factor that explains the greater prevalence and the gender gap. However, some studies indicate a later age of onset in women in most of the chronic morbidities analyzed. In the USA, a study on the age of onset of cardiovascular disease and its risk factors showed that women are diagnosed at a later age than men in hypercholesterolemia (51.6 vs. 48.7, respectively), hypertension (48.8 vs. 46.8), and coronary artery disease (59.3 vs. 57.4), with the exception of stroke, where women are diagnosed earlier (57.6 vs. 58.7) [59]. A larger study in England analyzing the age of onset of 308 physical and mental conditions showed that women are diagnosed later than men in most of the morbidities included in this study [60]. The difference in mean age at first diagnosis between men and women was generally less than 3 years. In the Canary Islands, women had a life expectancy 5 years longer than men in 2015. If the above-indicated onset pattern were reproduced in the Canary Islands, the net result would be that women would live more years exposed to diseases than men, explaining this apparent paradox of later onset of chronic morbidity and unfavorable gender gap in women.

It has been suggested that the differences in morbidity between men and women could be more related to a differential exposure to risk factors than to a differential vulnerability [61]. Exposure to differential risk factors, such as being overweight for arthritis or smoking for lung diseases, has been shown to be a source of the gender gap in some morbidities [56]. However, it has been suggested that part of the excess of musculoskeletal diseases in women could be explained by differences in vulnerability, finding a gender-specific association only in overweight women to explain pain in 10 anatomical regions and for older age to explain pain in the legs [12]. It has been argued that differences in health and chronic morbidity between men and women require multifactorial explanations, due to the influence of social determinants on health in addition to genetic and biological differences [21]. It has been shown that men and women are differentially exposed to these determinants, and that the pathways that lead to these structural (socioeconomic, age, social support, family arrangement), behavioral (smoking, drinking, physical activity, sedentariness, diet), and psychosocial (critical life events, stress, psychological resources) factors that influence health and morbidity are not only different for men and women, but also that women respond differently from men to these factors with a sex-specific response [12,21,62,63,64].

Our study provides a dynamic perspective of the gender gap, verifying the stability of the gap for musculoskeletal diseases, headaches, anxiety and depression, and peripheral vascular diseases, suggesting a gap reduction in high cholesterol, high blood pressure, and digestive diseases. Heart diseases, diabetes, and respiratory diseases did not show a consistent gender gap. This gap reduction may be due to a different trend in men and women in the prevalence of the self-reported chronic morbidities.

### 4.2. Trend in Chronic Morbidities

All chronic morbidities analyzed, except heart diseases in women and respiratory diseases in men, increased their crude ratio of change between 1997 and 2015. This increasing trend was largely explained by aging in the population. In women, the adjustment for age canceled out the crude increase trend in high blood pressure and respiratory diseases, while in men it canceled out the crude increase trend in respiratory diseases, heart diseases, headaches, joint–rheumatic pain, and osteoporosis. In the rest of the morbidities, both the *p*-value and the ratio of change decreased substantially, between 20–92% in women (between −0.005 and −0.026 annual OR) and between 22–91% in men (between −0.007 and −0.031 annual OR) after adjusting for age and education.

Despite this decrease in the trend rate found in the general population after adjusting for age group and education level, most of the morbidities analyzed maintained a significant increase in the rate of change in both genders. This finding is in alignment with previous research conducted at the national and regional levels of the trend (2006–2017) of 10 frequent chronic morbidities in Spanish adults >65 years [38], which shows an expansion (expressed as a decrease in the percentage of morbidity-free life expectancy) of hypertension, back pain, high cholesterol, and diabetes, with the exception of some cardiovascular diseases and respiratory chronic conditions. In our study, heart and respiratory diseases did not show a consistent increase trend after adjustment in both genders, which could be due to a decrease in smoking among younger cohorts. Smoking was the main risk factor of respiratory and cardiovascular diseases in Spain between 1990–2016 [37,65]. In Denmark, also, the stagnation of life expectancy in the 1980s and 1990s has been linked to lifestyle factors, especially smoking [66]. Nevertheless, in our study the aggregated cardiovascular diseases (second level) showed an increase trend in both genders, due to the rise in the trend of high cholesterol and peripheral vascular diseases in women, and that of high blood pressure, high cholesterol, and peripheral vascular diseases in men. Previous research has shown that a morbidity can present divergences in its compression or expansion tendency if it is defined as a major disease [24].

Spain has experienced a large decline in mortality in old age, and a much faster aging process in the demographic than in other countries in the European Union [67]. In 2017, at the age of 65, 90% of the initial generation was still alive, and at the age of 90, a third remained alive [68]. The expansion of morbidity in Spain (1990–2010) has been confirmed to advance faster in terms of overall life expectancy than in terms of healthy life expectancy [34], with the result that Spaniards live longer with a certain degree of morbidity as opposed to without. Our study did not directly analyze the expansion or compression of chronic morbidities, which would require estimating the trend of healthy as opposed to unhealthy life expectancy in relation to overall life expectancy [35], but it is consistent with an expansion of most of the morbidities analyzed in the general population, since after disregarding population aging, the increasing trend, albeit slower, was consistent in most morbidities. The analysis that included the total sample was not sufficient to determine whether the increasing trend was the same in all age groups. To examine this question we analyzed the interaction of age with time (1997–2015).

The results showed a different pattern in the trend of morbidities in men and women. In women, the group >60 years old, unlike the rest of the age groups, significantly increased its rate of change in most of the morbidities analyzed. In men, an earlier increase trend was observed for various morbidities in the 46–60 age group of men (neck–back pain, anxiety and depression, high cholesterol, peripheral vascular diseases, digestive diseases, diabetes, aggregated cardiovascular and aggregated musculoskeletal morbidities). The rest of the morbidities in men aged 46 to 60 and above also generally showed an increasing rate of change, but it was not significant. These results suggest that the increase in morbidity in the general population (>16 years) was mainly due to a faster increasing trend in old age groups, and earlier in men than in women.

There are no apparent reasons to explain the earlier trend pattern in men vs. women for high cholesterol, diabetes, digestive diseases, and peripheral vascular diseases. It could be due to an earlier onset of the disease in men. A Spanish study that used national data on hospitalizations (1997–2010) and which focused on cardiovascular diseases and some types of cancer failed to find evidence of compression when the age of onset was taken as a definition of compression for morbidity [37]. The age of onset for major cancers was advanced between 2 and 5 years, while in cardiovascular diseases it decreased very slightly. The age of onset for cardiovascular disease in women (between 58–64 years of age) was 4 to 10 years later than in men (between 54–59 years of age) [37]. In Catalonia (1994–2011), a similar pattern was observed for diabetes and hypertension, which increased substantially in men >50 years of age, while in women it increased in those >60, whilst heart disease increased substantially from the age of 60 in men and from the age of 70 in women [36].

When comparing the rate of increase in men and women, no significant differences were obtained; however, men showed a higher rate of change in high blood pressure, peripheral vascular diseases, high cholesterol, and digestive diseases, which would explain the reduction in the gender gap for these morbidities. Other studies in the Spanish population (1994–2011) have shown a faster progression in men for hypertension, diabetes, and high cholesterol [36]. In the rest of the morbidities (diabetes, respiratory diseases, neck–back pain, heart diseases, anxiety and depression, joint–rheumatic pain, headaches, and osteoporosis) the differences between the trend found in men and women were small and not significant, and similar for both genders. A national study that included the Canarian region also found an expansion (expressed as an increase in unhealthy life expectancy) for both genders, unlike other Spanish regions where compression was observed only in women [38]. In Catalonia (1994–2011), no gender differences were observed in the expansion trend of morbidity at the age of 65, similarly increasing the percentage of years of life with disease for both men and women (from 52 to 70% in men and 56 to 72% in women) [36]. These results suggest that in the future the burden of care of older people will increase, and that the expansion of morbidity, although more consistent in older age groups, may be occurring in all age groups. This implies that in the future more people would need health care at an earlier age. Future studies with larger samples and longitudinal designs could confirm the higher rate of increase of the main chronic morbidities in the older age groups.

The trend of increasing chronic morbidities in the older groups of men and women, after adjusting for age and educational level in our study, reflects the presence of additional factors that drive the increase trend in the older age groups. A greater awareness of health care services in the older population and better knowledge of the most prevalent morbidities in both the general population and the medical community could increase the number of self-reported diagnoses [69,70,71]. Silent diseases such as diabetes and hypertension are now diagnosed earlier, and are in turn receiving better treatment [72]. In England (1993–2013), it was shown that later-born cohorts have a tendency to report poor health at the same age, suggesting a secular trend towards poorer perceived health [33]. It cannot be ruled out that those over 60 years of age in 2015 reported more morbidities than those in 1997.

The increasing trend in chronic morbidities in older age groups is probably due to specific causes that require a particular analysis of concrete morbidity [73]. However, most of the morbidities analyzed in our study share a few exposure factors related to lifestyle that may explain the increasing trend in both genders. Smoking, low education, alcohol consumption, physical inactivity, and diet were found to be important risk factors with incidence in long-standing limiting illness and health expectancy in Denmark [66]. In Spain, obesity and smoking have been proposed as major risk factors to explain an earlier age of onset for most chronic conditions and an increase in the fraction of the remaining life spent with a chronic disease [37]. Obesity increased in Spain over the observational period [74,75]. Likewise, physical inactivity defined as zero physical activity (PA) of moderate to vigorous intensity (MVPA) increased in the Canarian region between 1997 and 2004, despite the slight increase in the recommended MVPA levels [76]. Those with zero MVPA showed independent associations with cholesterol disorders, diabetes, and high blood pressure. A sedentary lifestyle defined as sitting time could also be an independent risk factor that explains the increase in morbidity [77,78]. There are no data on a sedentary lifestyle trend in the Canary Islands, but, given the increasing exposure to screens and automated transport, sitting time may have also increased, in turn contributing to the increase in chronic morbidity [79,80].

The data indicate an increase and accumulation of chronic morbidities in older people who will require health care. The increase in population at old and very old ages will pose a challenge for health systems. The Canary Islands health system should take into account the allocation of resources to strengthen primary and secondary care services for the elderly. Prevention work is also important if we want a healthier population with less morbidity in the older people. The high relationship between some chronic diseases, such as hypertension, heart disease, and diabetes, contributes to increasing the risk of cardiovascular events and also exacerbates the effect of some risk factors such as obesity and smoking [81]. Many cardiovascular diseases are preventable and require a global approach for their prevention [82]. Some simple primary care strategies such as BMI monitoring could prevent diabetes and hypertension [83]. Musculoskeletal morbidities associated with age could be delayed and mitigated through the prescription of physical activity and education about its benefits [36]. Other strategies aimed at reducing the impact of known risk factors, such as smoking, physical inactivity, sitting time, poor diet, and alcohol consumption, are necessary to promote healthier lifestyles and delay the main chronic morbidities.

This study has some limitations that affect the results and, therefore, needs to be viewed with caution. The institutionalized population is not included in our analysis; thus, the results apply only to the community-dwelling population. Previous results in morbidity and disability studies, including the institutionalized population, did not affect the overall results [39]. The prevalence data are self-reported and for those noncommunicable morbidities, such as hypertension, diabetes, high cholesterol, and osteoporosis, an underestimation is highly probable, since such morbidities would not have yet been diagnosed. Some studies conducted in the general population indicate that 15–20% of the Canarian participants are unaware of having diabetes [84,85]. Similarly, unknown hypertension in adults over 18 years old in the Canary Islands could account for 33% of clinically diagnosed cases [85]. If the diagnostic capacity of the health system increases, unknown chronic morbidity tends to decrease, resulting in an increase in diagnosed chronic morbidity. The reported information on morbidities could be biased due to unequal access to health care, but in the Canary Islands there are no differences between islands and municipalities with regard to health care access and diagnosis. The public health system is universal and serves 99.9% of the population [86]. Likewise, access to specialists to obtain a diagnosis has not shown territorial and socioeconomic inequalities that could bias the data on the morbidities reported here [87].

On the other hand, gender differences depend on whether men and women answer the questions in a similar way. One study found that women reported less accurate information for diabetes and hypertension than men [88]. Other research has shown reasonably accurate estimates based on self-reported records when comparing them with medical records for diabetes, stroke, and myocardial infarction, without gender affecting the validity of self-reports for various cardiovascular morbidities [13,89,90]. An important variable in our study that could explain the increase in some musculoskeletal and metabolic morbidities, such as BMI, did not complete the full time series and could not be included in this study. Between 1993 and 2006, morbid obesity increased in Spain from 1.6 to 6.1 per thousand inhabitants, and it was more prevalent in women, with differences greater than double except for 2006. Men experienced a faster growth of morbid obesity (6% per year) than women (4% per year) [74]. Finally, the data are representative of the Canarian population, which is mostly Caucasian and shares genes with the Spanish and Southern European population [91]. Its generalizability is limited to the Canarian population and could be relevant to other Spanish and European regions with similar age and gender profiles. Its generalizability to other different populations (e.g., Asians, Africans) could be limited.

## 5. Conclusions

Musculoskeletal diseases, headaches, anxiety and depression, and peripheral vascular diseases showed a stable gender gap across observed years. High cholesterol, high blood pressure, and digestive diseases tended to a gap reduction, whilst heart disease, diabetes, and respiratory disease did not show a significant gender gap along the period. Most of the morbidities showed an interaction with age in the analyzed period, evincing a faster increasing trend in the older age groups. Men presented an earlier pattern of increase in various chronic morbidities, compared to women. The trend of the main chronic morbidities increased similarly in men and women in all age groups, significantly in women older than 60 years of age and in men older than 45. Aging explained a substantial part of the trend of increasing prevalence of the main chronic morbidities, but not totally. Factors other than age and education are driving the increase in chronic morbidity in older age groups.

## Figures and Tables

**Table 1:** Characteristics of the participants. Canary Health Survey (1997, 2004, 2009, and 2015).

	Women				Men			
	1997	2004	2009	2015	1997	2004	2009	2015
	(*n* = 1124)	(*n* = 2513)	(*n* = 2655)	(*n* = 2591)	(*n* = 1043)	(*n* = 1791)	(*n* = 1887)	(*n* = 1969)
	%	%	%	%	%	%	%	%
Age								
16–30	30.6	20.3	15.5	12.3	32.1	23.0	18.5	12.8
31–45	27.8	29.3	30.6	25.0	28.1	31.0	33.0	28.7
46–60	20.1	19.4	24.2	29.7	22.0	20.1	22.7	29.7
>60	21.5	31.0	29.8	32.9	17.8	25.9	25.8	28.8
Mean age (years)	43.1	48.4	49.4	51.9	41.8	46.3	47.2	50.2
Education ^a^								
Primary or lower	47.3	33.4	32.9	26.1	43.5	32.0	28.2	21.2
High school	39.6	43.8	43.0	44.2	42.5	47.3	47.1	50.1
University	13.1	17.5	23.9	29.3	14.0	19.2	24.6	28.3
Occupation ^a^								
Employed	32.7	35.1	34.3	40.9	63.6	53.8	46.5	50.5
Unemployed	7.0	11.9	20	23.6	9.9	10.7	20.1	23.2
Student	9.7	6.0	4.0	6.8	9.7	6.8	4.0	4.6
Housework	31.2	17.8	17.2	6.1	0.2	0.7	0.2	0.2
Retired	16.3	28.3	22.4	30.4	19.4	26.7	26.5	29.1

**^a^** Standardized by age following the direct method, taking as reference the age and gender structure of the Canary population ≥ 16 years old in 2015.

**Table 2:** Differences between men and women in selected chronic morbidities. Canary Health Survey, 1997–2015.

Prevalence Ratio (95% CI) ^a^								
	1997		2004		2009		2015	
Musculoskeletal (2nd level) ^b^	**1.95 (1.70; 2.23)**	*^,a–c^	**2.30 (2.11; 2.51)**	*^,b^	**2.06 (1.94; 2.19)**	*^,a,c^	**1.90 (1.81; 2.08)**	*^,a,c^
Cardiovascular (2nd level) ^c^	**1.27 (1.02; 1.51)**	*^,a^	**1.31 (1.08; 1.36)**	*^,a^	**1.28 (1.09; 1.47)**	*^,a^	**1.29 (1.19; 1.40)**	*^,a^
Osteoporosis	**3.18 (1.66; 6.09)**	*^,a^	**3.69 (2.38; 5.73)**	*^,a^	**3.67 (2.46; 5.46)**	*^,a^	**3.59 (2.44; 5.21)**	*^,a^
Joint–rheumatic pain	**1.98 (1.67; 2.29)**	*^,a^	**2.07 (1.89; 2.26)**	*^,a^	**2.03 (1.88; 2.19)**	*^,a^	**2.24 (2.08; 2.40)**	*^,a^
Peripheral vascular disease	**2.30 (1.87; 2.74)**	*^,a–c^	**2.56 (2.21; 2.94)**	*^,b^	**1.67 (1.40; 1.99)**	*^,c^	**1.94 (1.71; 2.15)**	*^,a,c^
Chronic headaches	**1.72 (1.23; 2.41)**	*^,a^	**1.74 (1.40; 2.17)**	*^,a^	**1.79 (1.47; 2.16)**	*^,a^	**1.88 (1.57; 2.29)**	*^,a^
Neck and back pain	**1.79 (1.43; 2.23)**	*^,a^	**1.75 (1.51; 2.02)**	*^,a^	**1.40 (1.19; 1.61)**	*^,a^	**1.86 (1.68; 2.05)**	*^,a^
Anxiety, depression	**1.48 (1.14; 1.92)**	*^,a^	**1.40 (1.17; 1.68)**	*^,a^	**1.62 (1.43; 1.81)**	*^,a^	**1.57 (1.38; 1.73)**	*^,a^
Digestive diseases	1.22 (0.94; 1.58)	^a^	**1.35 (1.13; 1.66)**	*^,a^	**1.37 (1.18; 1.59)**	*^,a^	1.18 (0.98; 1.39)	^a^
High cholesterol	1.21 (0.95; 1.49)	^a^	**1.23 (1.02; 1.45)**	*^,a^	1.16 (0.99; 1.31)	^a^	1.12 (0.98; 1.26)	^a^
High blood pressure	1.28 (0.99; 1.57)	^a^	1.19 (0.98; 1.40)	^a^	1.03 (0.87; 1.19)	^a^	1.01 (0.89; 1.16)	^a^
Respiratory diseases	1.15 (0.92; 1.43)	^a^	1.14 (0.95; 1.32)	^a^	1.21 (0.98; 1.44)	^a^	1.17 (0.98; 1.37)	^a^
Diabetes	1.14 (0.81; 1.59)	^a^	1.21 (0.98; 1.44)	^a^	1.16 (0.95 1.41)	^a^	1.16 (0.97; 1.36)	^a^
Heart diseases	0.85 (0.65; 1.03)	^a^	0.88 (0.75; 1.14)	^a^	0.96 (0.78; 1.19)	^a^	0.90 (0.71; 1.19)	^a^

^a^ Age-standardized prevalence of women/age-standardized prevalence of men. ^b^ At least one morbidity including chronic neck and back pain, joint–rheumatic pain, and osteoporosis. ^c^ At least one morbidity including high blood pressure, heart disease, peripheral vascular disease, high cholesterol, and stroke. * *p* < 0.05 (χ^2^ test) for prevalence ratio (highlighted in bold). A superscript letter represents significant differences between years (*p* < 0.05, Holm–Bonferroni test), a different letter expresses significant differences, and the same letter expresses no significant differences.

**Table 3:** Rate of change in 1997–2015 of the analyzed chronic morbidity for the overall sample and age groups.

		Overall (*n* = 8884)					Age Groups				
		Crude		Adjusted		16–30 years old (*n* = 1585)	31–45 years old (*n* = 2510)	46–60 years old (*n* = 2126)		>60 years old (*n* = 2664)	
**nº**	**Women**	OR (95% IC) **^a^**		OR (95% IC) **^b^**		OR (95% IC) **^c^**	OR (95% IC) **^c^**	OR (95% IC) **^c^**		OR (95% IC) **^c^**	
	Musculoskeletal (2nd level)	**1.043 (1.035; 1.051)**	***	**1.020 (1.007; 1.033)**	*	1.018 (0.981; 1.053)	1.017 (0.991; 1.043)	1.019 (0.991; 1.047)		**1.035 (1.010; 1.060)**	**
	Cardiovascular (2nd level)	**1.046 (1.038; 1.054)**	***	**1.026 (1.012; 1.040)**	**	1.016 (0.980; 1.050)	1.019 (0.989; 1.049)	1.024 (0.996; 1.052)		**1.045 (1.021; 1.069)**	***
1	Neck–back pain	**1.043 (1.035; 1.052)**	***	**1.019 (1.003; 1.035)**	*	1.012 (0.976; 1.048)	1.014 (0.977; 1.041)	1.015 (0.986; 1.044)		**1.033 (1.007; 1.059)**	*
2	Anxiety Depression	**1.037 (1.028; 1.045)**	***	**1.026 (1.012; 1.040)**	**	1.021 (0.985; 1.057)	1.014 (0.985; 1.044)	**1.034 (1.002; 1.066)**	*	**1.041 (1.013; 1.069)**	**
3	High blood pressure	**1.028 (1.019; 1.037)**	***	1.002 (0.987; 1.017)		0.982 (0.943; 1.027)	0.984 (0.956; 1.012)	1.005 (0.973; 1.037)		**1.039 (1.014; 1.055)**	**
4	High cholesterol	**1.039 (1.027; 1.047)**	***	**1.016 (1.002; 1.030)**	*	1.007 (0.971; 1.045)	1.009 (0.984; 1.035)	1.025 (0.995; 1.055)		**1.024 (1.001; 1.047)**	*
5	Joint–rheumatic pain	**1.031 (1.022; 1.041)**	***	**1.015 (1.001; 1.029)**	*	0.987 (0.948; 1.023)	0.995 (0.969; 1.021)	1.014 (0.983; 1.045)		**1.037 (1.002; 1.073)**	*
6	Peripheral vascular disease	**1.044 (1.032; 1.056)**	***	**1.018 (1.002; 1.035)**	*	1.018 (0.979; 1.048)	1.019 (0.989; 1.049)	1.022 (0.989; 1.055)		**1.029 (1.001; 1.057)**	*
7	Digestive diseases	**1.036 (1.025; 1.047)**	***	**1.019 (1.004; 1.034)**	*	0.999 (0.961; 1.037)	1.010 (0.980; 1.045)	1.023 (0.991; 1.055)		**1.031 (1.003; 1.059)**	*
8	Headaches	**1.039 (1.030; 1.048)**	***	**1.021 (1.008; 1.033)**	*	1.030 (0.995; 1.065)	1.027 (0.998; 1.056)	1.020 (0.987; 1.053)		1.015 (0.988; 1.042)	
9	Diabetes	**1.024 (1.013; 1.035)**	**	**1.019 (1.003; 1.035)**	*	1.011 (0.973; 1.050)	1.012 (0.984; 1.030)	1.021 (0.988; 1.055)		**1.031 (1.004; 1.058)**	*
10	Respiratory diseases	**1.036 (1.024; 1.048)**	**	1.012 (0.994; 1.030)		1.018 (0.974; 1.058)	1.010 (0.979; 1.041)	1.012 (0.977; 1.047)		1.015 (0.986; 1.044)	
11	Osteoporosis	**1.047 (1.034; 1.060)**	***	**1.028 (1.009; 1.049)**	**	---	1.021 (0.988; 1.055)	1.027 (0.989; 1.065)		**1.035 (1.003; 1.067)**	*
12	Heart diseases	1.011 (0.999; 1.023)		1.005 (0.988; 1.022)		0.993 (0.952; 1.034)	1.007 (0.973; 1.038)	1.009 (0.973; 1.045)		1.021 (0.982 1.061)	
**nº**	**Men**	Overall (*n* = 6690)				16–30 years old (*n* = 1350)	31–45 years old (*n* = 2038)	46–60 years old (*n* = 1601)		>60 years old (*n* = 1701)	
	Musculoskeletal (2nd level)	**1.035 (1.026; 1.044)**	***	**1.025 (1.010; 1.041)**	**	1.018 (0.980; 1.056)	1.019 (0.991; 1.046)	**1.031 (1.002; 1.059)**	*	**1.036 (1.008; 1.066)**	*
	Cardiovascular (2nd level)	**1.047 (1.038; 1.056)**	***	**1.022 (1.006; 1.038)**	**	1.011 (0.974; 1.038)	1.020 (0.993; 1.047)	**1.034 (1.004; 1.065)**	*	**1.037 (1.008; 1.066)**	*
3	Neck–back pain	**1.043 (1.032; 1.054)**	***	**1.017 (1.001; 1.033)**	*	1.012 (0.973; 1.051)	1.005 (0.971; 1.035)	**1.036 (1.003; 1.069)**	*	1.029 (0.998; 1.060)	
5	Anxiety Depression	**1.041 (1.030; 1.052)**	***	**1.023 (1.006; 1.041)**	*	1.021 (0.982; 1.060)	1.019 (0.987; 1.051)	**1.040 (1.006; 1.074)**	*	1.028 (0.996; 1.061)	
1	High blood pressure	**1.038 (1.026; 1.050)**	***	**1.028 (1.010; 1.046)**	*	1.015 (0.974; 1.056)	1.023 (0.990; 1.055)	1.031 (0.995; 1.067)		**1.045 (1.010; 1.080)**	**
2	High cholesterol	**1.055 (1.044; 1.067)**	***	**1.024 (1.007; 1.041)**	*	1.009 (0.969; 1.042)	1.019 (0.979; 1.049)	**1.034 (1.001; 1.067)**	*	**1.042 (1.009; 1.075)**	*
9	Joint–rheumatic pain	**1.024 (1.012; 1.036)**	**	1.010 (0.991; 1.029)		1.005 (0.967; 1.047)	1.011 (0.977; 1.045)	1.008 (0.969; 1.047)		1.012 (0.962; 1.052)	
7	Peripheral vascular disease	**1.059 (1.045; 1.073)**	***	**1.031 (1.011; 1.052)**	**	1.021 (0.978; 1.064)	1.013 (0.979; 1.047)	**1.051 (1.013; 1.089)**	**	**1.045 (1.008; 1.082)**	*
4	Digestive diseases	**1.044 (1.031; 1.057)**	***	**1.027 (1.009; 1.045)**	**	1.024 (0.984; 1.063)	1.020 (0.989; 1.021)	**1.038 (1.003; 1.073)**	*	1.033 (0.998; 1.066)	
10	Headaches	**1.039 (1.027; 1.051)**	***	1.014 (0.997; 1.031)		1.032 (0.995; 1.069)	1.018 (0.985; 1.051)	1.015 (0.979; 1.051)		0.993 (0.957; 1.028)	
6	Diabetes	**1.039 (1.026; 1.052)**	***	**1.020 (1.002; 1.039)**	*	0.973 (0.934; 1.021)	1.014 (0.982; 1.046)	**1.036 (1.001; 1.071)**	*	**1.045 (1.009; 1.081)**	*
11	Respiratory diseases	1.009 (0.995; 1.023)		1.011 (0.988; 1.030)		0.997 (0.946; 1.048)	1.009 (0.971; 1.043)	1.029 (0.992; 1.066)		1.021 (0.985; 1.057)	
12	Osteoporosis	**1.022 (1.006; 1.038)**	*	1.015 (0.987; 1.037)		---	1.012 (0.974; 1.050)	1.013 (0.972; 1.054)		1.025 (0.985; 1.065)	
8	Heart diseases	**1.024 (1.013; 1.037)**	**	1.002 (0.982; 1.022)		0.991 (0.938; 1.045)	1.015 (0.981; 1.049)	1.005 (0.966; 1.044)		1.022 (0.983; 1.061)	

^a^ Odds ratio (OR) per year of the trend of prevalence 1997–2015 for the overall sample. ^b^ OR per year adjusted by age and education. ^c^ OR per year adjusted by age, education, and the interaction age group x time. *** *p* < 0.001; ** *p* < 0.01; and * *p* < 0.05 for the OR. nº = order of prevalence in 2015. Highlighted in bold is the rate of change with *p* < 0.05.

## Data Availability

The original data are available to researchers upon request to Instituto Canario de Estadística, e-mail Consultas.istac@gobiernodecanarias.org. Microdata of variables used in this study are available in the Zenodo repository. The 1997 dataset CHS is at https://doi.org/10.5281/zenodo.5653080; the 2004 dataset CHS is at https://doi.org/10.5281/zenodo.5653156; the 2009 dataset CHS is at https://doi.org/10.5281/zenodo.5653175; the 2015 dataset CHS is at https://doi.org/10.5281/zenodo.5653217, all accessed on 28 June 2022.

## Supplementary Materials

The following supporting information can be downloaded at: https://www.mdpi.com/article/10.3390/ijerph19159404/s1, Figure S1. Evolution of the population structure of age, gender and education in Canary between 1997 and 2015.
