# Supplementary material for: Stable Gender Gap and Similar Gender Trend in Chronic Morbidities between 1997–2015 in Adult Canary Population
Source: Int J Environ Res Public Health. 2022 Jul 31;19(15):9404. doi: 10.3390/ijerph19159404 (PMC9368162; doi:10.3390/ijerph19159404)
Supplement: Supplementary file 1 [file ijerph-19-09404-s001.zip › ijerph-1815544-supplementary.pdf]

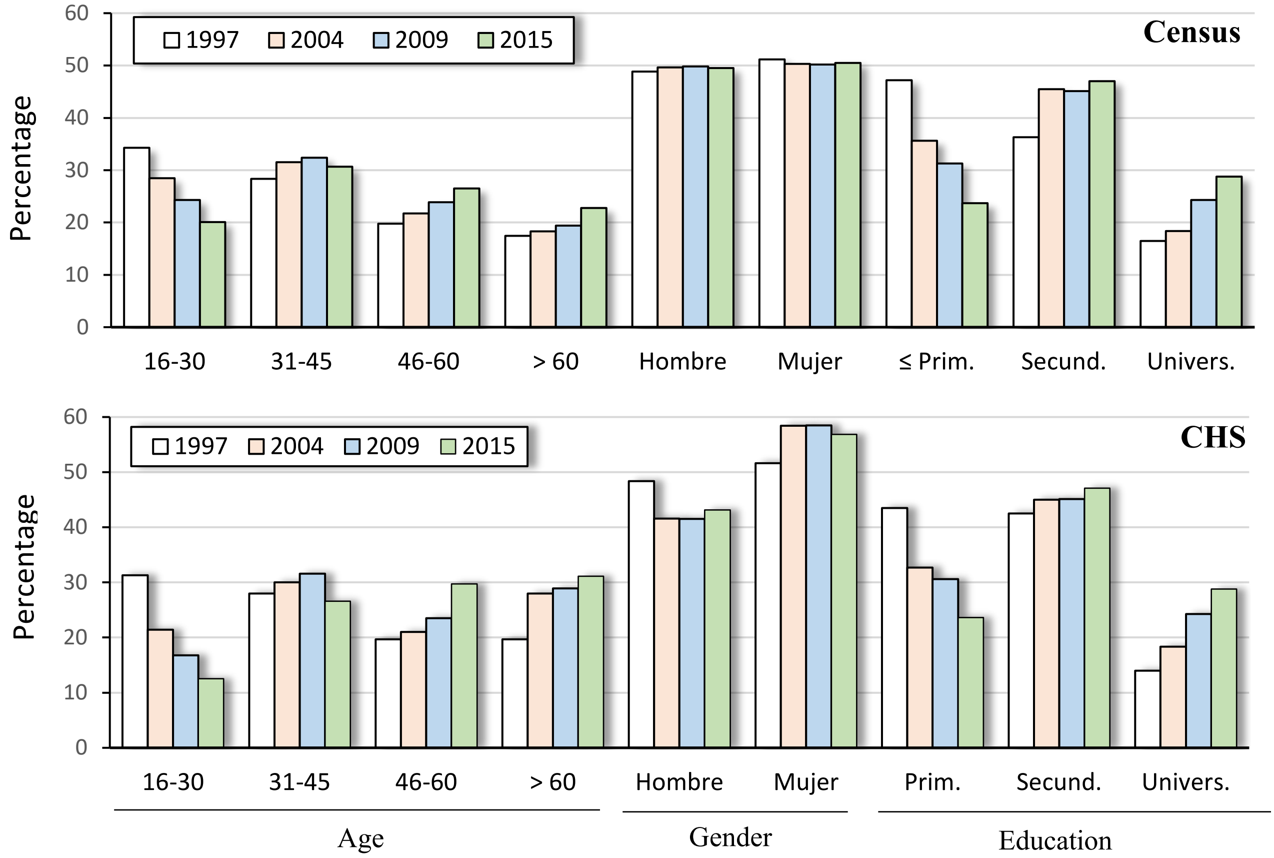

FIgure S1. Evolution of the population structure of age, gender and education in Canary between 1997 a 2015. Above, census data, below, data from the Canary Islands Health Survey

and
